# Fascial Closure vs. Non-closure of Right Working Port Sites in Laparoscopic Bariatric Surgery: A Randomized Clinical Trial

**DOI:** 10.1007/s11695-025-07917-2

**Published:** 2025-05-23

**Authors:** Mohamed Yacoub, George A. Nashed, Ahmed Y. Khalifa, Ahmed M. Hassan

**Affiliations:** Cairo University, Giza, Egypt

**Keywords:** Bariatric surgery, Fascial closure, Obesity, Port site bleeding, Port site hernia, Port site infection, Randomized clinical trial

## Abstract

**Background:**

Laparoscopic surgery offers benefits like reduced bleeding, pain, and shorter hospital stays but poses port-site complications, especially in patients with obesity. Postoperative pain is less severe than in open surgery. Multimodal analgesia is promising, while the impact of fascial closure on complications remains debated. This study aims to compare the incidence of port-site complications in patients undergoing laparoscopic bariatric surgery, with or without fascial closure of the right working port.

**Methods:**

This randomized clinical trial was reported based on the CONSORT checklist. Seventy patients with severe obesity were compared in terms of fascial closure versus non-closure of the right working port during laparoscopic bariatric surgery. Thorough clinical, radiological, and nutritional assessment was done. Postoperative pain (using VAS) and port-site complications were assessed. Ethical approval was obtained, and informed consent was guaranteed.

**Results:**

Patients who underwent fascial closure of the right working port demonstrated significantly higher rates of moderate (85.7% vs. 5.7%) and severe pain (14.3% vs. 2.9%, *p* < 0.001) compared to the non-closure group. Port-site complications—bleeding (8.6% vs. 2.9%, *p* = 0.303), infection (11.4% vs. 14.3%, *p* = 0.721), and hernia (11.4% vs. 2.9%, *p* = 0.178)—were statistically comparable, suggesting that fascial closure may increase postoperative pain without significant impacts on other complications.

**Conclusions:**

Fascial closure of the right working port after laparoscopic bariatric surgery in patients with obesity increases postoperative pain without reducing port-site complications like bleeding, infection, or herniation. Non-closure appears safer and effective in minimizing pain and adverse outcomes. Routine fascial closure may not be justified, warranting further research to optimize surgical techniques for patients with obesity.

## Introduction

Along with the benefits of laparoscopic approaches in various surgical procedures, comes significant challenges. Although laparoscopic surgery mitigates perioperative bleeding and postoperative pain, providing brief hospital stays, among other advantages [1], port-site adverse events such as bleeding, herniation, and infection, persist [2].

The incidence of port-site complications following laparoscopic surgery varies reported to be around 0.23–1.9% for common trocar sizes used in various laparoscopic procedures, rising proportionally to the size of the trocar [2, 3]. Bariatric surgery is not exempt from this; in fact, patients with obesity are at higher risk of port-site complications [4]. Higher body weight is a significant risk factor for port-site complications due to specific surgical considerations, such as the need for trocars of greater sizes, larger incisions to accommodate anatomical variations, and potential limitations in instrument mobility due to increased adipose tissue [5]. Additionally, individuals with obesity may experience impaired wound healing compared to other populations [6].

Laparoscopic bariatric surgery results in less postoperative pain compared to open bariatric surgery [7], facilitated by the reduced trauma to the abdominal wall and smaller incisions in laparoscopic procedures [3]. Nevertheless, intense pain has been reported in the first 24 h postoperatively, progressively decreasing over the following days [8, 9], although transient, postoperative pain is major concern when considering the quality of life of patients [10].

To address postoperative pain, the use of multimodal analgesia was proposed [11], aiming to enhance pain relief while minimizing the risk and severity of side effects [12]. Common strategies include combining intravenous analgesics with epidural analgesia or injecting local anesthetics at the port site [13, 14].

Other novel techniques have been postulated to curtail postoperative complications, such as the use of biological plugs instead of conventional fascial suturing; however, the debate on whether to perform port-site fascial closure after laparoscopic surgery remains unresolved [1, 15]. Many authors suggested that closure must be done when large-sized trocars are utilized [16]; however, this remains unclear in the context of bariatric surgeries. In addition, no consensus has been reached regarding the impact of fascial closure on postoperative complications such as pain, bleeding, infection, and port-site herniation [17, 18].

In the following randomized clinical trial, we evaluated the impact of fascial closure on the outcomes of bariatric surgery, with special emphasis on postoperative pain and port-site complications in patients with obesity.

## Methods

### Trial Design

**Fig. 1:**
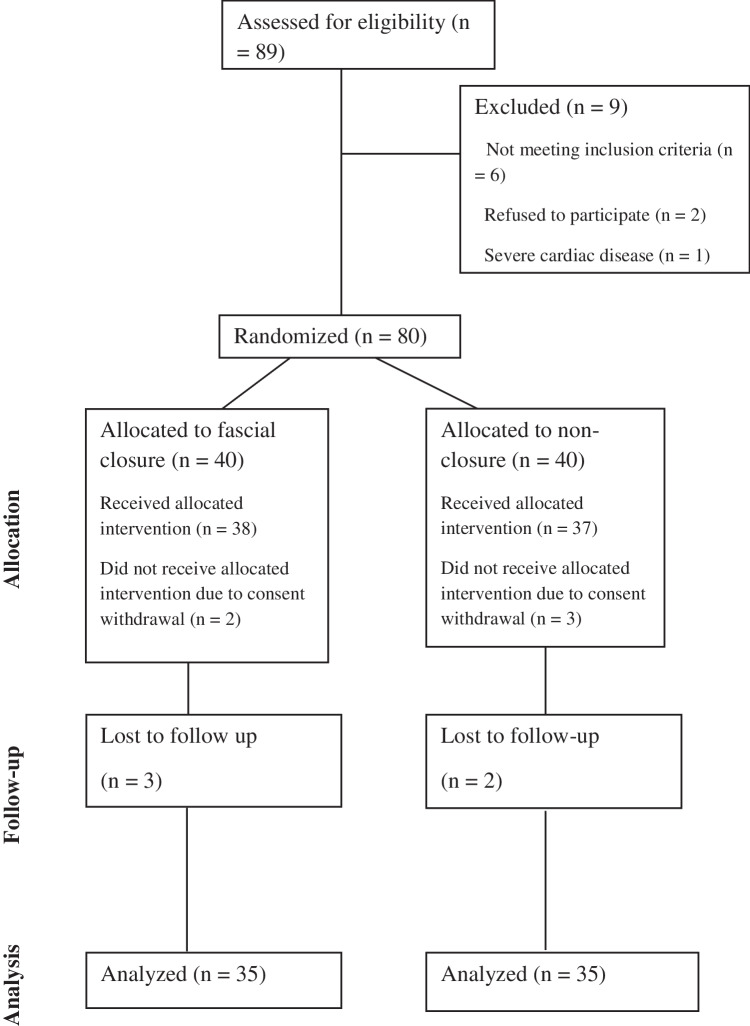
CONSORT diagram showing the flow of participants through each stage of the trial

In the following single-blinded randomized clinical trial, our reporting adhered to the tenets of the CONSORT checklist [19]. We included a group of 70 patients with severe obesity, who were candidates for laparoscopic bariatric surgery, randomly allocating them in blocks of four using a computer-generated randomization schedule, at a 1:1 ratio into two groups, to maintain balance throughout enrollment (Fig. 1). The closure group (*n* = 35) received fascial closure of the right working port, whereas the non-closure group (*n* = 35) did not.

### Sample Size

The sample size was calculated with the software G-Power using Fisher’s exact test to compare the rate of postoperative pain under a two-tailed hypothesis, which was derived from data presented in a similar previous work (p1 = 0.119 vs. p2 = 0.659) [1]. With a strict power requirement of 99% (*β* = 0.01) and an alpha-level set at 0.05, the analysis determined that 30 participants per group (total = 60) would suffice to detect this large effect size. However, we included 40 patients per group (total = 80) in case dropouts occurred during the study period, and our final analysis was conducted on 35 patients per group (total = 70). The exact method yielded an actual power of 99.23% and an actual *α* of 0.026. The 1:1 allocation ratio ensured balanced group sizes.

### Participants

This trial was carried out over a period of 6 months at Kasr Al-Ainy Hospital, Cairo University (April–October 2024). At the time of surgery, we included patients who were above the age of 14, with class II–III obesity, or class I with significant associated medical conditions, patients who experienced suboptimal clinical response to conservative/medical options—such as diet, exercise, and/or obesity management medications (OMMs) for at least 6 months, as well as patients who were accepting of surgical risks. We excluded patients who underwent previous abdominal exploration or prior bariatric surgery, as well as those with preexisting nutritional deficiencies, significant longstanding cardiorespiratory disease or severe systemic illness, as well as those with ongoing pregnancy or lactation, active gastric ulcer disease, active substance or alcohol intake, and individuals experiencing significant psychological distress.

### Interventions

A complete preoperative evaluation was ensured for all patients, including thorough history-taking, physical examination, routine laboratory tests, an endocrinological workup, chest x-ray, pulmonary function tests, as well as full cardiological assessment and abdominal ultrasound. Moreover, all patients were referred to a dietician for preoperative and postoperative nutritional counselling.

Prior to the operation, all patients were placed on a low-calorie diet for a period of 1–3 weeks, according to their baseline BMI, and all associated medical conditions were controlled enough to safeguard participants’ wellbeing. Moreover, after informing our participants about the surgery, we described the procedural modifications being studied (fascial closure vs. non-closure), taking care not to suggest that one method was preferable to the other. Since patients were blinded to the procedure they received, we minimized the risk of their pain perception being influenced by prior expectations or biases.

**Fig. 2:**
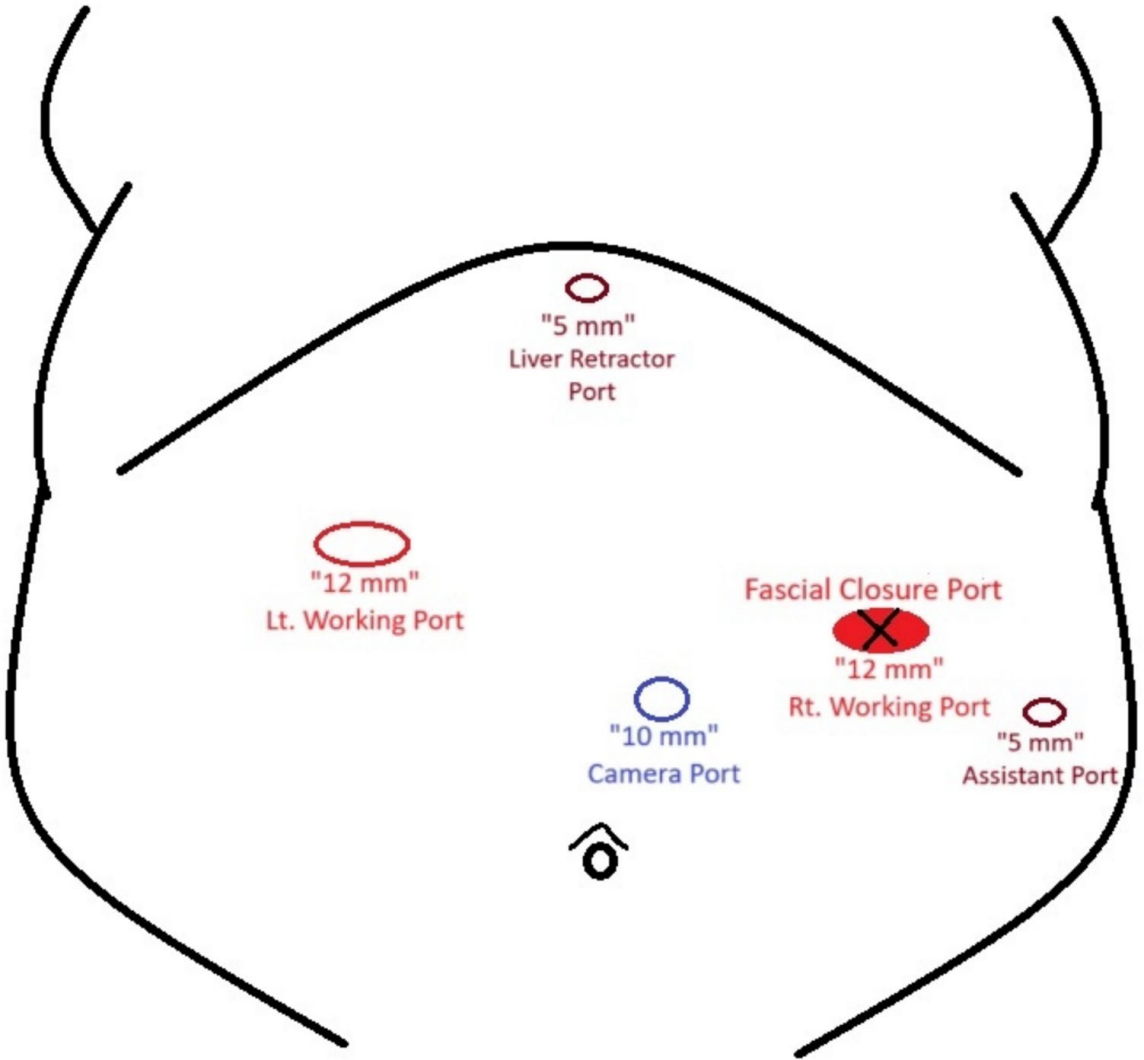
Diagram illustrating the right working port where fascial closure or non-closure was performed

**Fig. 3:**
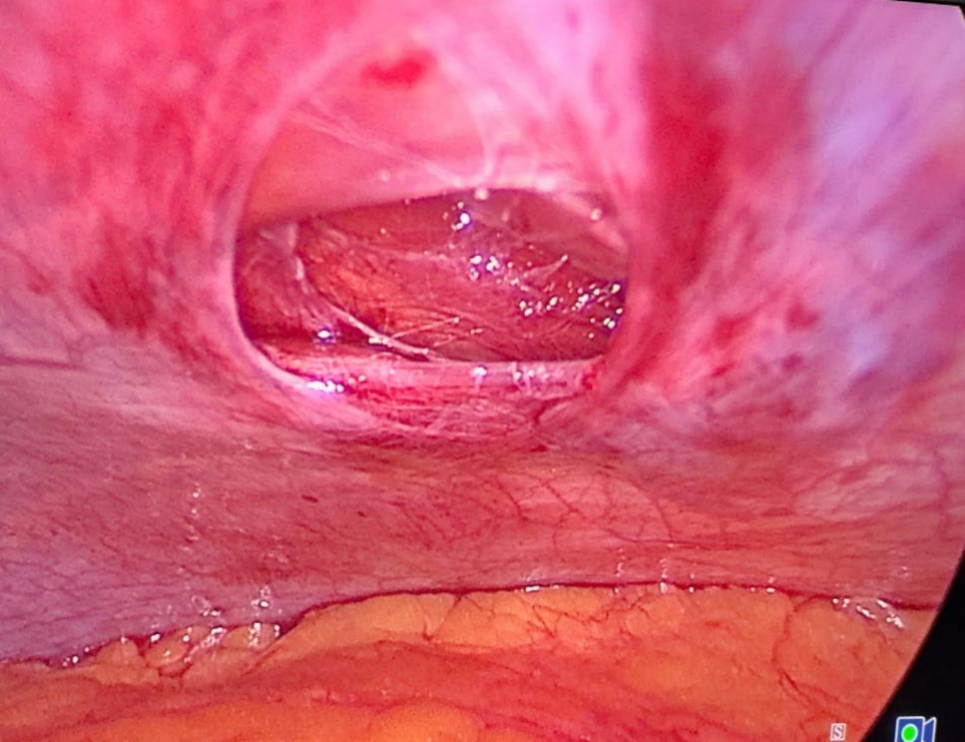
Right working port before fascial closure

**Fig. 4:**
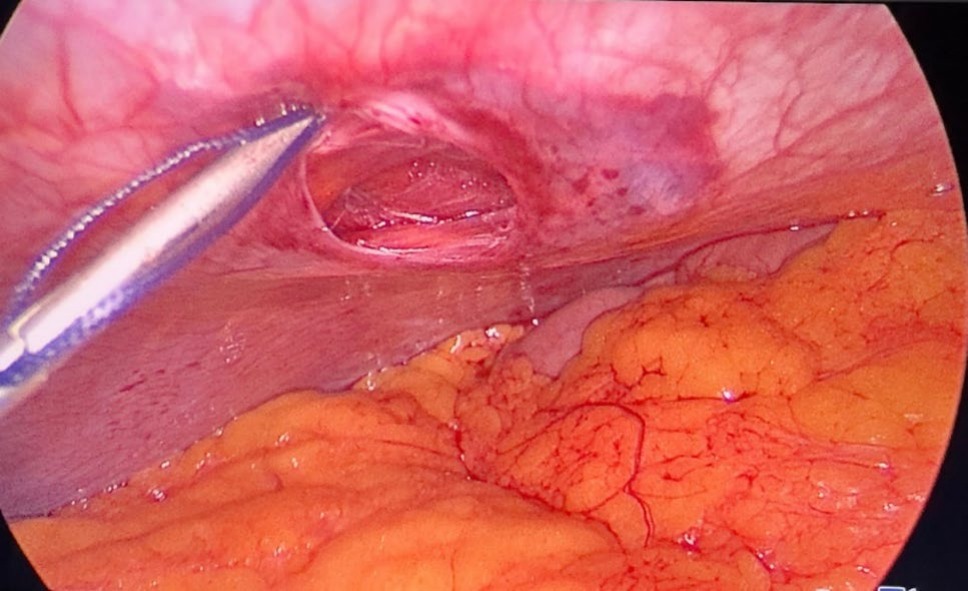
The Endoclose device “Berci needle” preloaded with the Vicryl 1 suture inserted between the skin and the port edge

**Fig. 5:**
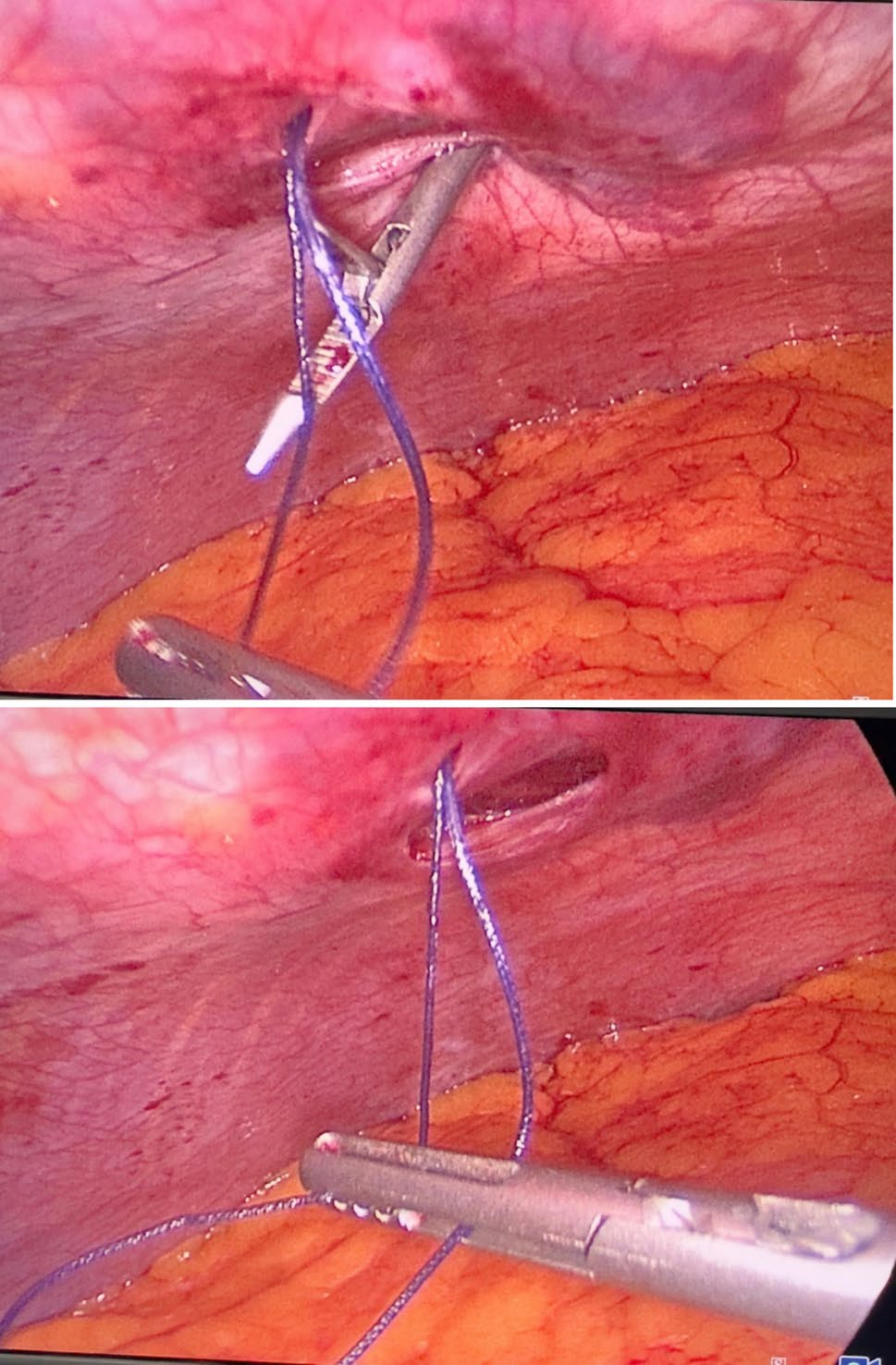
A 5-mm grasping forceps retrieving the Vicryl 1 suture from the Endoclose device

**Fig. 6:**
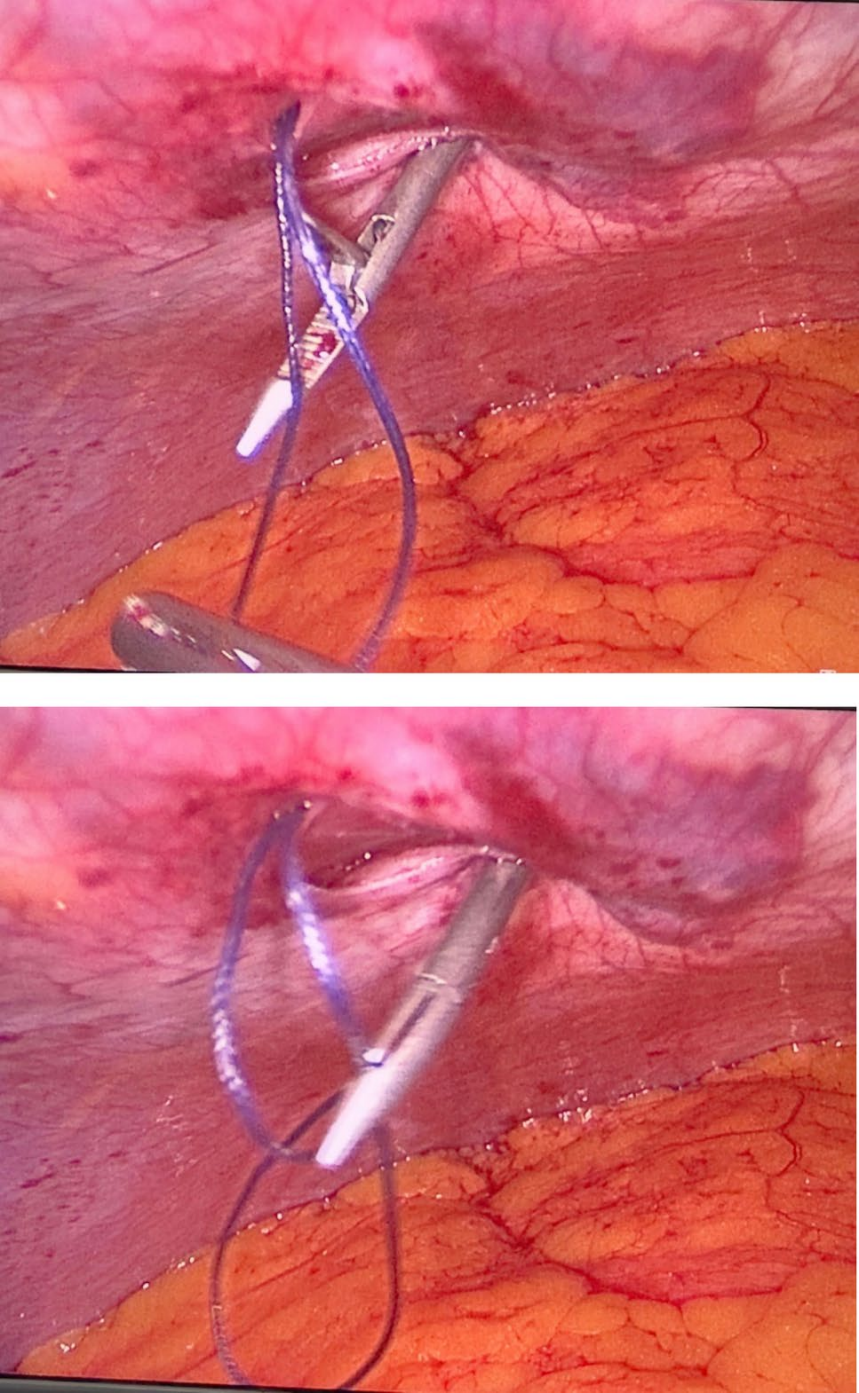
Reloading of the Vicryl 1 suture onto the Endoclose device

**Fig. 7:**
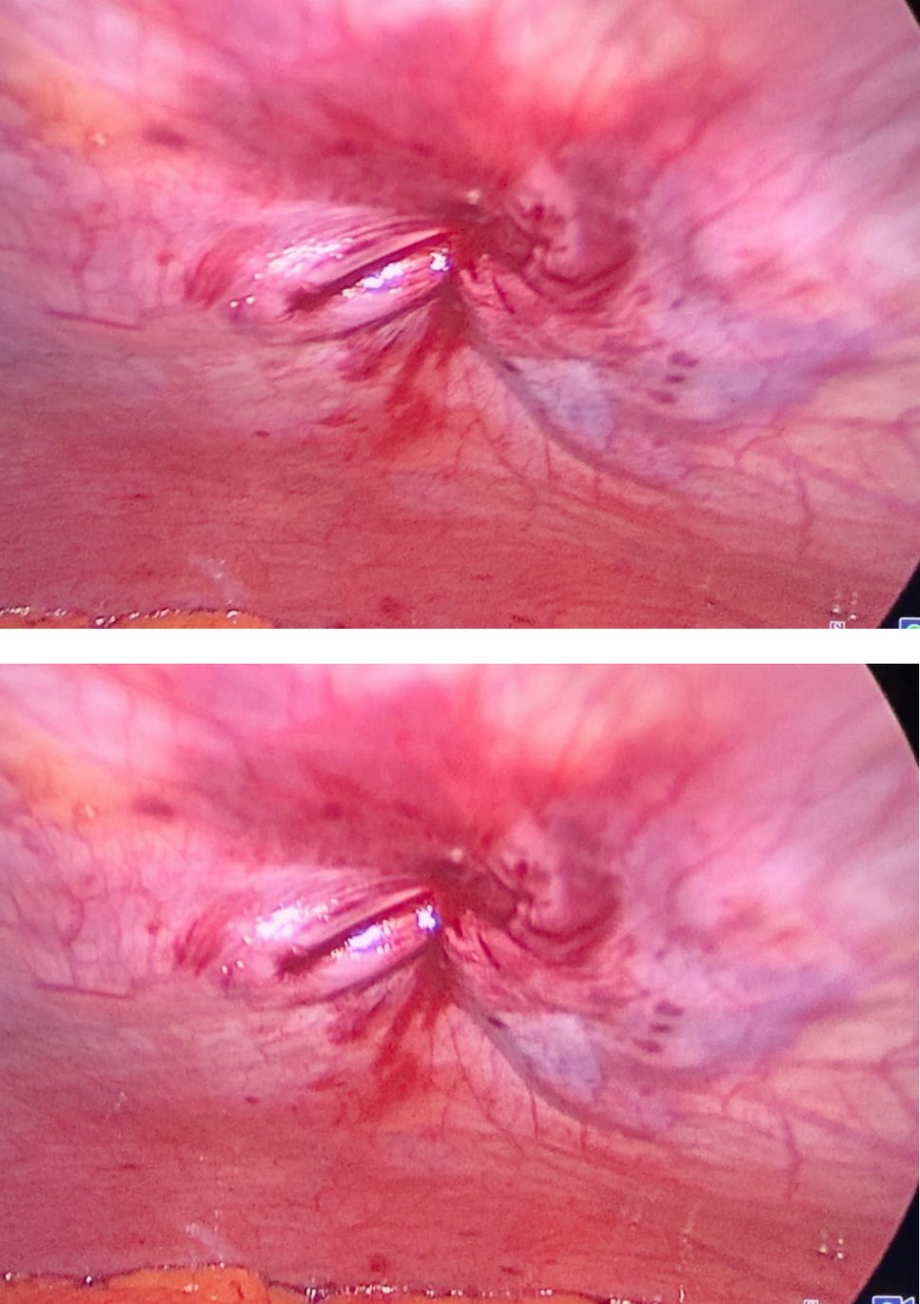
Fascial defect closure after Vicryl 1 suture exteriorization

- *Surgery*: Laparoscopic procedures were performed using a 10-mm camera port, two 12–15-mm operating ports, a 5-mm Nathanson liver retractor, and a 5-mm assistant port. CO_2_ insufflation was maintained at 14 mmHg. One group underwent fascial closure at the right working port using a Vicryl 1 suture with an Endoclose device (Fig. 2), while the other group had no suturing (Fig. 3). Postoperative care protocols were identical for all patients.
- *Anesthetic Protocol*: Patients received balanced general anesthesia with standard monitoring—3-lead ECG, SpO₂, non-invasive blood pressure (NIBP), and neuromuscular monitoring. Invasive arterial monitoring was reserved for unreliable NIBP. Positioned in reverse Trendelenburg, induction included 2 mg/kg fentanyl as well as 0.5 mg/kg atracurium to facilitate tracheal intubation. Maintenance involved 1–1.2% isoflurane in oxygen-air mixture and 0.1 mg/kg atracurium every 20 min. At the beginning of the operation, all patients received dexamethasone 8 mg unless they were on insulin or oral hypoglycemic medications. At the end of the operation, parecoxib 40 mg, droperidol 0.625 mg, and ondansetron 4 mg were given. Paracetamol 2 g was administered on arrival to post-anesthesia care unit (PACU), with antiemetics and opioids (oxycodone or fentanyl) administered as needed. Postoperative continuous SpO₂ monitoring was established.
- *Technique*: A disposable Endoclose device preloaded with Vicryl 1 suture was inserted between the skin and port edge (Fig. 4). The suture was deployed intra-abdominally, and the device withdrawn. The suture carrier was then passed 180° opposite the insertion site, through fascia and peritoneum. A 5-mm grasping forceps (via a secondary port) retrieved the suture, reloading it onto the Endoclose device (Figs. 5 and 6). The suture was exteriorized and tied to close the fascial defect (only in the closure group; Fig. 7).

### Study Outcomes

- *Primarily*: postoperative pain was assessed by PACU nursing staff and recorded using the visual analogue scale (VAS) [20], at 2 and 4 h, postoperatively.
- *Secondarily*: clinical and ultrasonographic assessment of port-site infection, bleeding, and hernia in the first 24 h and after 6 months of the operation.

### Statistical Methods

All data were analyzed using the Statistical Package for the Social Sciences (SPSS), version 28. The normality of data distribution was tested using the Kolmogorov–Smirnov single-sample test. Qualitative data were presented as frequencies and percentages, with associations between groups analyzed using the Chi-square test. Quantitative variables were expressed as mean ± standard deviation (SD) for normally distributed data. Continuous variables were compared between the two groups using the independent *T*-test. A *p*-value of ≤ 0.05 was considered statistically significant for all analyses.

## Results

Our study involved 70 participants who were randomly divided into two equal groups of 35 each. One group underwent the closure technique, while the other group underwent the non-closure technique.

The sociodemographic characteristics of patients are shown in Table 1. In both groups, the age distribution was comparable, averaging 33.3 years in the non-closure group and 32.3 years in the closure group (*p* = 0.672; Table 1). In terms of gender, a female preponderance was evident at a rate of 85.7% in the non-closure group, and 77.1% in the closure group; however, no statistically significant differences were present (*p* = 0.356; Table 1).

**Table 1:** Sociodemographic characteristics of participants

	Non-closure group (*n* = 35)	Closure group (*n* = 35)	*p* value
Age (years)	33.3 ± 10.0	32.3 ± 9.7	0.672
Gender			0.356
Female	30 (85.7%)	27 (77.1%)	
Male	5 (14.3%)	8 (22.9%)	

In terms of relevant medical and surgical history, 25.71% of our patients had chronic illnesses, and with no statistically significant difference between either group (28.6% vs. 22.9%, *p* = 0.584; Table 2). Among those chronic illnesses, diabetes mellitus was the most notable at rate of 60% in the non-closure group, and 25% in the closure group (*p* = 0.138; Table 2). In addition, the surgical history of patients is presented in Table 2, and cesarean sections are the most reported in both groups (57.8% vs. 72.7%, *p* = 0.424; Table 2). As shown, the mean BMI was similar in the non-closure and the closure groups (47.4 vs. 48.2 kg/m^2^; *p* = 0.510; Table 2), and most patients fell in class III obesity. Most patients in both groups underwent sleeve operation at an equal rate of 82.9%, and the operative time, while slightly longer for the closure group, did not significantly differ from that in the non-closure group (121 vs. 115 min, *p* = 0.069; Table 2).

**Table 2:** Medical, surgical history, BMI, type of surgery, and operative time among the participants

History	Non-closure group (*n* = 35)	Closure group (*n* = 35)	*p* value
Chronic disease			0.584
No	25 (71.4%)	27 (77.1%)	
Yes	10 (28.6%)	8 (22.9%)	
Type of chronic disease			0.138
Diabetes	6 (60%)	2 (25%)	
Hypertension	2 (20%)	4 (50%)	
Hypothyroidism	1 (10%)	1 (12.5%)	––-
Surgical history			0.053
No	16 (45.7%)	24 (68.6%)	
Yes	19 (54.3%)	11 (31.4%)	
Type of previous surgery			
Hernioplasty	0 (0%)	2 (18.2%)	0.058
Tonsillectomy	3 (15.7%)	1 (9.1%)	0.609
Cesarean	11 (57.8%)	8 (72.7%)	0.424
Appendectomy	4 (21.1%)	2 (18.2%)	0.852
BMI	47.4 ± 5.4	48.2 ± 5.5	0.510
Type of operation			1.000
Bypass	6 (17.1%)	6 (17.1%)	
Sleeve	29 (82.9%)	29 (82.9%)	
Operative time (M ± SD)	115 (± 12)	121 (± 15)	0.069

N.B: Ten patients from the non-closure group and eight patients from the closure group had chronic illnesses. Of these, only diabetes, hypertension, and hypothyroidism were deemed relevant to the analysis and explicitly mentioned. Similarly, 19 patients in the non-closure group reported prior surgeries, but only the four most reported procedures were listed (hernioplasty, tonsillectomy, Cesarean section, and appendectomy)

Pain was the most reported postoperative complication. In comparison to the non-closure group, the closure group had significantly higher rates of moderate (85.7% vs. 5.7%) and severe pain (14.3% vs. 2.9%, *p* < 0.001; Table 3). On the other hand, there were no statistically significant differences between the closure and non-closure groups in terms of port-site bleeding (2.9% vs. 8.6%, *p* = 0.303; Table 3), port-site infection (14.3% vs. 11.4%, *p* = 0.721; Table 3), or port-site hernia (2.9% vs. 11.4%, *p* = 0.178; Table 3).

**Table 3:** Postoperative complications among the participants

	Non-closure group (*n* = 35)	Closure group (*n* = 35)	*p* value
Pain score			< 0.001*
Mild	32 (91.4%)	0 (0%)	
Moderate	2 (5.7%)	30 (85.7%)	
Severe	1 (2.9%)	5 (14.3%)	
Port site bleeding			0.303
No	32 (91.4%)	34 (97.1%)	
Yes	3 (8.6%)	1 (2.9%)	
Port site infection			0.721
No	31 (88.6%)	30 (85.7%)	
Yes	4 (11.4%)	5 (14.3%)	
Port site hernia			0.178
No	31 (88.6%)	34 (97.1%)	
Yes	4 (11.4%)	1 (2.9%)	

## Discussion

This randomized clinical trial aimed to compare the incidence of port-site complications in patients with obesity who underwent laparoscopic bariatric surgery with or without fascial closure.

We estimated the mean operative time for each group and determined that, although fascial suturing was associated with a slightly longer operative time, the difference in comparison to the non-closure group was not statistically significant (121 vs. 115 min, *p* = 0.069); however, considering that 17.1% of patients in each group underwent gastric bypass, which requires a significantly longer duration to perform compared to sleeve gastrectomy [21]; the analysis might have overestimated the mean operative time for each group. This was in line with Andraos who stated that omitting fascial suturing resulted in less operative time, but the significance of this was not revealed as they concealed their operative details [1]. Nevertheless, prolonged operative times in bariatric surgery are related to more perioperative complications and a higher mortality rate. Morino et al. revealed that operative time during bariatric surgery was a significant risk factor for perioperative mortality, demonstrating that those who died due to the procedure had significantly longer operative time compared to those who remained alive (183 vs. 112 min, *p* < 0.05) [22]. Inaba et al. reported a mean operative time of 78 min in laparoscopic sleeve gastrectomy (LSG), and 104 min in gastric bypass (LRYGB). Their analysis revealed that for every 10-min increase in operative time, the odds of postoperative complications at the 1-year follow-up rose significantly for LSG (OR 1.07, *p* = 0.0002) and LRYGB (OR 1.03, *p* < 0.001). Moreover, mortality increased with an OR of 1.04, for every additional 10 min in LRYGB (*p* = 0.02). These findings confirm that prolonged operative duration adversely impacts both complication rates and mortality following laparoscopic bariatric surgery [23].

We noted that pain was the most common postoperative complication for all patients. In the non-closure group, 91.4% of patients experienced only mild postoperative pain. Conversely, in the closure group, 85.7% reported moderate pain and 14.3% reported severe pain, compared with 5.7% and 2.9%, respectively, in the non-closure group, and this difference was statistically significant (*p* < 0.001). Consistent with our findings, Andraos revealed that traditional suturing techniques for fascial closure after laparoscopic bariatric surgery were associated with significantly higher early postoperative pain as opposed to using a biological plug of absorbable gelatin without suturing (65.9% vs. 11.9%, *p* < 0.001). Moreover, upon follow-up, no chronic pain was reported in the open fascia group compared to 12.2% of those who received fascial closure (*p* = 0.026) [1]. Similarly, although under a divergent protocol, Sonbol et al. inferred that fascial closure after laparoscopic hernial repair was associated with substantially higher median postoperative pain as evidenced by exceedingly higher VAS scores compared to repair without primary fascial closure (3 vs. 2, *p* = 0.031) [24]. Contrastingly, Jawed et al. advocated for the use of Endostitch fascial closure device as it was associated with less postoperative pain scores compared to the traditional port site closure technique (2.279 vs. 3.346, *p* < 0.05). They explained these findings by highlighting that the Endostitch device avoided full-thickness bites during fascial closure, which prevented the inadvertent suturing of subcutaneous fat and muscle layers [25]. However, these findings were contrasted by Lyapis et al. who revealed that the fascial closure device, in fact, increased pain levels on the first postoperative day [26]. Coblijn et al. inferred that fascial closure following bariatric surgery in individuals with obesity does not lower the incidence of port-site complications; on the contrary, it can result in nerve constriction which is a notable cause of postoperative pain [18]. This hypothesis of neuralgia was in line with a report by Kellegrew et al. who described the occurrence of ilioinguinal/iliohypogastric nerve entrapment with associated significant postoperative pain following a laparoscopic surgery with fascial closure. Pain relief was definitive after the release of fascial sutures. In contrast, postoperative neuralgia was not an issue in patients who underwent laparoscopic surgeries without fascial closure. Given the routine insertion of the laparoscopic trocars above the level where the ilioinguinal and iliohypogastric nerves course [27], they postulated that smaller trocars without subsequent fascial suturing would prevent such injuries, resulting in less postoperative pain [28].

We evaluated the incidence of port-site bleeding in our patients and demonstrated an overall rate of 5.7%, with no statistically meaningful differences between the two groups (*p* = 0.303). Andraos indicated that leaving the fascia at the port site open and using Cutanplast, a biological hemostatic plug, demonstrated no incidence of trocar site bleeding, unlike traditional suturing which resulted in bleeding in 12.5% of patients (*p* = 0.024). They also showed that fascial closure was associated with considerably higher rate of hematoma formation (29.3% vs. 2.4%, *p* = 0.001) [1]. Hubballi et al. emphasized the correlation between obesity and port-site bleeding by comparing patients with obesity to those with an average or below average BMI (6.9% vs. 0% vs. 0%, *p* = 0.0411). Intriguingly, all their patients underwent fascial closure with traditional suturing [4].

In our study, port-site infection was not a prominent postoperative complication in either group (*p* = 0.721), and the overall estimated incidence was 12.6%. Andraos reported in line with our findings, demonstrating no statistically meaningful difference in port-site infection whether fascial closure was undertaken or not (9.8% vs. 0%, *p* = 0.055) [1]. Hubballi et al. noted that in patients with obesity who received fascial closure following laparoscopic surgery, the rate of port-site infection was considerably higher in comparison to other patients whose weight was much lower (*p* = 0.0402) [4]. On the other hand, Pilone et al., who implemented a non-closure technique in bariatric surgery, reported no incidence of port-site infection over a follow-up period of 54 months, corroborating the superiority of this technique in mitigating postoperative complications [29].

We reported no incidence of early port-site hernia 24 h after the operation. On the other hand, with an overall incidence of 7.1%, port-site hernia occurred 6 months after the operation at a comparable rate between those who received fascial closure and those who did not (2.9% vs. 11.4%, *p* = 0.178). In accordance with our findings, Gutierrez et al. followed up on patients who underwent bariatric surgery over a period of 22 months and observed no statistically significant difference in the incidence of port-site herniation, whether fascial closure was done or not. They compared the incidence of open versus closed techniques in relation to the size of the trocar used and found that both 5-mm ports (0.08% vs. 0%, *p* = 1.000) and 10-mm ports (0.19% vs. 0.14%, *p* = 0.634) were similar in this regard [30]. Chiu et al. described a notably low incidence of port-site herniation (0.33%) after a mean follow-up of 29 months in patients who underwent bariatric surgery without fascial closure [15]. Conversely, Andraos observed exceedingly higher incidence of port-site hernia in bariatric surgeries with fascial suturing, at a rate of 17.1% as opposed to none of the patients whose port-site was merely closed with a biological plug (*p* = 0.005) [1]. Pilone et al., who deployed a non-closure technique for the fascia, investigated larger port sizes and found that only ten patients (1.6% of their overall sample) developed port-site hernias, three of those required a 15-mm port as opposed to 10 mm in the other seven [29]. These findings suggest that port size may not be the only factor contributing to port-site hernias in patients with obesity and other patient-related or technical factors may be involved. In their analysis, Coblijn et al. supported this hypothesis by demonstrating an overall prevalence of 0.94% of port-site herniation in various laparoscopic bariatric surgeries. Upon further analysis, they highlighted that closure of the fascia of the port site led to a higher incidence of port-site herniation especially in patients with obesity, an inverse correlation that was not seen in patients with a lower BMI [4]. They deduced that this could be attributed to technical issues associated with difficult trans-fascial suturing in this population of patients. Alternatively, tissue ischemia was also implicated. Although their recommendation was to omit fascial closure of the port site—as it resulted in no benefit and more complications, they acknowledged the scant evidence to confirm or deny this recommendation in the context of bariatric surgery [18].

Despite achieving high statistical power, the following study is limited by its relatively small sample size and single-center design. These limitations highlight the need for larger, multicenter trials with longer follow-up to validate our findings.

## Conclusion

In conclusion, our study highlighted that fascial closure of the right working port following laparoscopic bariatric surgery in patients with obesity is associated with significantly higher postoperative pain compared to non-closure techniques, without providing meaningful benefits in reducing port-site complications such as bleeding, infection, or herniation. The findings align with previous studies which suggested that fascial closure may contribute to nerve entrapment, leading to increased pain and other adverse outcomes. Conversely, omitting fascial closure appears to be a safer and equally effective approach, particularly in mitigating postoperative pain and complications, with a less significant impact on operative time. While port-site herniation remains a concern, its incidence does not significantly differ between closure and non-closure groups, and other factors such as trocar size and patient-specific variables may play a more critical role. Based on the evidence, the routine use of fascial closure in laparoscopic bariatric surgery may not be justified, and further research is warranted to refine surgical techniques and optimize outcomes for patients with obesity.

## Data Availability

Data supporting the findings of our trial will be provided upon request from the corresponding author.

## References

[1] Andraos Y. Safety and efficacy of trocar port-site closure using a biological plug closure in laparoscopic bariatric surgery: a prospective study. Obes Surg. 2022;32(11):3796–806. 36071329 10.1007/s11695-022-06238-y PMC9613580

[2] Hotston M, et al. Port site hernias following robot-assisted laparoscopic prostatectomy. J Robot Surg. 2009;3(1):49–51. 27628454 10.1007/s11701-009-0133-y

[3] Maharaul HH, Jain N, Garg P. Port site complications following laparoscopic surgeries. Int J Surg Sci. 2019;3(3):318–24.

[4] Hubballi K, Togale MD, Hubballi BG. Are we neglecting port-site complications in laparoscopic surgeries: a single centre study. Med Sci. 2023;27(132):1–10.

[5] Sasmal PK, et al. Port site infection in laparoscopic surgery: a review of its management. World J Clin Cases. 2015;3(10):864–71. 26488021 10.12998/wjcc.v3.i10.864 PMC4607803

[6] Cotterell A, et al. Understanding wound healing in obesity. World J Exp Med. 2024;14(1):86898. 38590299 10.5493/wjem.v14.i1.86898 PMC10999071

[7] Nguyen NT, Varela JE. Bariatric surgery for obesity and metabolic disorders: state of the art. Nat Rev Gastroenterol Hepatol. 2017;14(3):160–9. 27899816 10.1038/nrgastro.2016.170

[8] Inan A, Sen M, Dener C. Local anesthesia use for laparoscopic cholecystectomy. World J Surg. 2004;28(8):741–4. 15457350 10.1007/s00268-004-7350-3

[9] Zapf M, et al. Patient-centered outcomes after laparoscopic cholecystectomy. Surg Endosc. 2013;27:4491–8. 23943114 10.1007/s00464-013-3095-0

[10] Wu CL, et al. The effect of pain on health-related quality of life in the immediate postoperative period. Anesth Analg. 2003;97(4):1078–85. 14500161 10.1213/01.ANE.0000081722.09164.D5

[11] Schumann R, et al. A comparison of multimodal perioperative analgesia to epidural pain management after gastric bypass surgery. Anesth Analg. 2003;96(2):469–74. 12538198 10.1097/00000539-200302000-00032

[12] American Society of Anesthesiologists Task Force on Acute Pain Management. Practice guidelines for acute pain management in the perioperative setting: an updated report by the American Society of Anesthesiologists Task Force on Acute Pain Management. Anesthesiology. 2012;116(2):248–273. 10.1097/ALN.0b013e31823c1030 22227789

[13] Cantore F, et al. Pre-incision local infiltration with levobupivacaine reduces pain and analgesic consumption after laparoscopic cholecystectomy: a new device for day-case procedure. Int J Surg. 2008;6:S89–92. 19264565 10.1016/j.ijsu.2008.12.033

[14] Bertin PM. Liposome bupivacaine for postsurgical pain in an obese woman with chronic pain undergoing laparoscopic gastrectomy: a case report. J Med Case Rep. 2014;8(1):21. 24450503 10.1186/1752-1947-8-21 PMC3974146

[15] Chiu CC, et al. Prevention of trocar-wound hernia in laparoscopic bariatric operations. Obes Surg. 2006;16(7):913–8. 16839493 10.1381/096089206777822269

[16] Kadar N, et al. Incisional hernias after major laparoscopic gynecologic procedures. Am J Obstet Gynecol. 1993;168(5):1493–5. 8498433 10.1016/s0002-9378(11)90787-x

[17] Tonouchi H, et al. Trocar site hernia. Arch Surg. 2004;139(11):1248–56. 15545574 10.1001/archsurg.139.11.1248

[18] Coblijn UK, et al. Trocar port hernias after bariatric surgery. Obes Surg. 2016;26(3):546–51. 26164327 10.1007/s11695-015-1779-3

[19] Schulz KF, Altman DG, Moher D. CONSORT 2010 statement: updated guidelines for reporting parallel group randomized trials. Ann Intern Med. 2010;152(11):726–32. 20335313 10.7326/0003-4819-152-11-201006010-00232

[20] Jensen MP, Chen C, Brugger AM. Interpretation of visual analog scale ratings and change scores: a reanalysis of two clinical trials of postoperative pain. J Pain. 2003;4(7):407–14. 14622683 10.1016/s1526-5900(03)00716-8

[21] Kang DW, et al. Predicting operative time for metabolic and bariatric surgery using machine learning models: a retrospective observational study. Int J Surg. 2024;110(4):1968–74. 38270635 10.1097/JS9.0000000000001107 PMC11019972

[22] Morino M, et al. Mortality after bariatric surgery: analysis of 13,871 morbidly obese patients from a national registry. Ann Surg. 2007;246(6):1002–7; discussion 1007-9. 18043102 10.1097/SLA.0b013e31815c404e

[23] Inaba CS, et al. Operative time as a marker of quality in bariatric surgery. Surg Obes Relat Dis. 2019;15(7):1113–20. 31128998 10.1016/j.soard.2019.04.010

[24] Sonbol HA, et al. Comparative study between primary fascial closure versus non closure in laparoscopic ventral hernia repair with mesh. Egypt J Surg. 2025;44(1):213–9.

[25] Jawed AE, et al. A novel method of laparoscopic port site closure to significantly decrease postoperative surgical site pain - SAGES abstract archives. SAGES, 2016.

[26] Lyapis A, et al. Does the difference in fascial closure technique affect postoperative pain? J Minim Invasive Gynecol. 2017;24(7):1190–4. 28757438 10.1016/j.jmig.2017.07.020

[27] Whiteside JL, et al. Anatomy of ilioinguinal and iliohypogastric nerves in relation to trocar placement and low transverse incisions. Am J Obstet Gynecol. 2003;189(6):1574–8. 14710069 10.1016/s0002-9378(03)00934-7

[28] Kellegrew J, et al. Diagnostic and therapeutic ilioinguinal and iliohypogastric nerve blocks: a case report. A&a Pract. 2024;18(1): e01740. 10.1213/XAA.0000000000001740 38259135

[29] Pilone V, et al. Trocar site hernia after bariatric surgery: our experience without fascial closure. Int J Surg. 2014;12:S83–6. 24862661 10.1016/j.ijsu.2014.05.047

[30] Gutierrez M, et al. Does closure of fascia, type, and location of trocar influence occurrence of port site hernias? A literature review. Surg Endosc. 2020;34(12):5250–8. 32728766 10.1007/s00464-020-07826-8

